# Assessing the Radiation Response of Lung Cancer with Different Gene Mutations Using Genetically Engineered Mice

**DOI:** 10.3389/fonc.2013.00072

**Published:** 2013-04-02

**Authors:** Bradford A. Perez, A. Paiman Ghafoori, Chang-Lung Lee, Samuel M. Johnston, Yifan Li, Jacob G. Moroshek, Yan Ma, Sayan Mukherjee, Yongbaek Kim, Cristian T. Badea, David G. Kirsch

**Affiliations:** ^1^Department of Radiation Oncology, Duke University Medical CenterDurham, NC, USA; ^2^Department of Pharmacology and Cancer Biology, Duke University Medical CenterDurham, NC, USA; ^3^Department of Radiology, Center for In vivo Microscopy, Duke University Medical CenterDurham, NC, USA; ^4^Department of Statistical Science, Institute for Genome Sciences and Policy, Duke UniversityDurham, NC, USA; ^5^Department of Computer Science, Institute for Genome Sciences and Policy, Duke UniversityDurham, NC, USA; ^6^Department of Mathematics, Institute for Genome Sciences and Policy, Duke UniversityDurham, NC, USA; ^7^Department of Clinical Pathology, College of Veterinary Medicine, Seoul National UniversitySeoul, South Korea

**Keywords:** tumor cell biology, genetically engineered mouse models, fractionation, p53

## Abstract

**Purpose:** Non-small cell lung cancers (NSCLC) are a heterogeneous group of carcinomas harboring a variety of different gene mutations. We have utilized two distinct genetically engineered mouse models of human NSCLC (adenocarcinoma) to investigate how genetic factors within tumor parenchymal cells influence the *in vivo* tumor growth delay after one or two fractions of radiation therapy (RT).

**Materials and Methods:** Primary lung adenocarcinomas were generated *in vivo* in mice by intranasal delivery of an adenovirus expressing Cre-recombinase. Lung cancers expressed oncogenic Kras^G12D^ and were also deficient in one of two tumor suppressor genes: p53 or Ink4a/ARF. Mice received no radiation treatment or whole lung irradiation in a single fraction (11.6 Gy) or in two 7.3 Gy fractions (14.6 Gy total) separated by 24 h. In each case, the biologically effective dose (BED) equaled 25 Gy_10_. Response to RT was assessed by micro-CT 2 weeks after treatment. Quantitative reverse transcription-polymerase chain reaction (qRT-PCR) and immunohistochemical staining were performed to assess the integrity of the p53 pathway, the G1 cell-cycle checkpoint, and apoptosis.

**Results:** Tumor growth rates prior to RT were similar for the two genetic variants of lung adenocarcinoma. Lung cancers with wild-type (WT) p53 (LSL-Kras; Ink4a/ARF^FL/FL^ mice) responded better to two daily fractions of 7.3 Gy compared to a single fraction of 11.6 Gy (*P* = 0.002). There was no statistically significant difference in the response of lung cancers deficient in p53 (LSL-Kras; p53^FL/FL^ mice) to a single fraction (11.6 Gy) compared to 7.3 Gy × 2 (*P* = 0.23). Expression of the p53 target genes p21 and PUMA were higher and bromodeoxyuridine uptake was lower after RT in tumors with WT p53.

**Conclusion:** Using an *in vivo* model of malignant lung cancer in mice, we demonstrate that the response of primary lung cancers to one or two fractions of RT can be influenced by specific gene mutations.

## Introduction

A substantial body of clinical and radiobiological evidence suggests that small doses of fractionated radiation therapy (RT), in comparison with a single or a few high-dose treatments, may confer a therapeutic ratio because non-tumor tissues are preferentially spared (Hall and Giaccia, 2006). It is postulated that the radiobiological behavior of tumors (and normal tissues) is reflected in the α/β ratio that characterizes their radiation survival curves (Hall and Giaccia, 2006). However, the α/β ratio of a tumor is an empirically derived parameter and, therefore, the biological basis for differential tumor response to fractionated RT is largely unknown. Using isogenic derivatives of glioblastoma U87 cells (ATCC# HTB-14), some investigators have determined that the wild-type (WT) tumor suppressor gene p53 promotes radiosensitivity to fractionated RT (Gupta et al., 1996; Haas-Kogan et al., 1996, 1999; Yount et al., 1996, 1998) by maintaining the integrity of the cell-cycle G1-checkpoint (independent of apoptosis). However, others found no significant difference in clonogenic cell survival when comparing glioblastoma cells with WT (U87 cells) or mutant p53 (T98 cells, ATCC# CRL-1690) (Quick and Gewirtz, 2006). Non-small lung cancer cell lines have also been evaluated for evidence of cell-cycle synchronization following anti-cancer therapy prior to delivery of RT (Asaka-Amano et al., 2007). We wished to further investigate the role of an intact G1 cell-cycle checkpoint on the response of primary lung cancers *in vivo* to a single-dose or two fractions of RT using two different mouse models of primary non-small cell lung cancer (NSCLC).

A methodological approach for studying radiation biology *in vivo* using primary lung cancers in mice has been previously described (Kirsch et al., 2010a). Tumors in these genetically engineered mouse models (GEMMs) bear close resemblance to human tumors at the histological and genetic levels (Sweet-Cordero et al., 2005) and develop in a native tumor microenvironment within an immunocompetent mouse. Thus, these mouse models may have significant advantages for studying mechanisms of tumor response to RT. In addition to closely recapitulating human lung cancers (Sharpless and Depinho, 2006), GEMMs provide the opportunity to generate lung cancers harboring different gene mutations that are relevant to human lung cancer. In the present work, we utilize two distinct GEMMs of primary lung adenocarcinomas initiated by the conditional, Cre-mediated activation of oncogenic Kras^G12D^ (Jackson et al., 2001), and deletion of a tumor suppressor gene, either p53 (Jackson et al., 2005) or Ink4a^p16^/ARF^p19^ (Aguirre et al., 2003). p53 and Ink4a^p16^/ARF^p19^ have been shown to play critical roles in multiple cellular responses after exposure to RT, including cell-cycle arrest, senescence, and apoptosis (Gudkov and Komarova, 2003). Furthermore, along with Kras, these two tumor suppressor genes are known to be among the most frequently mutated genes in human lung adenocarcinomas (Ding et al., 2008). Using an *in vivo* approach, we describe differences in primary lung tumor response to fractionated RT based on mutations in p53 or Ink4a/ARF.

## Materials and Methods

The Duke Institutional Animal Care and Use Committee approved all of the described protocols using vertebrate animals.

### Mouse breeding

All mice expressed a conditional Lox-Stop-Lox *Kras^G12D/^+* (*LSL-Kras*) allele (Jackson et al., 2001). Expression of oncogenic *Kras^G12D^* is controlled by a Cre-regulatable transcriptional Stop element. Because this conditional oncogenic *Kras* allele was targeted into the endogenous locus (Tuveson et al., 2004), endogenous levels of oncogenic Kras^G12D^ protein are expressed following removal of the stop element upon expression of Cre-recombinase. *LSL-Kras* mice for this study were bred with mice with conditional alleles for the tumor suppressors p53 (Jonkers et al., 2001) or Ink4a/ARF (Aguirre et al., 2003). In these conditional alleles, LoxP sites flank important coding regions in these tumor suppressor genes and are termed floxed alleles: *p53^FL^* or *Ink4a/ARF^FL^*. These conditional alleles are targeted to their respective endogenous loci so that prior to deletion, expression of the respective WT tumor suppressor gene is normal. However, in the presence of Cre-recombinase the LoxP sites recombine, thereby creating a deletion with loss of tumor suppressor gene function. Mice were bred to contain one allele of the conditional oncogenic Kras gene while simultaneously containing tumor suppressor genes *p53* (*LSL-Kras; p53^FL/FL^*) or *Ink4a/ARF* (*LSL-Kras; Ink4a/ARF^FL/FL^*) with both alleles flanked by loxP sites.

### Infection with adenovirus expressing Cre-recombinase

Adult mice expressing the conditional alleles described above were infected with adenovirus expressing Cre-recombinase (Adeno-Cre) intranasally as previously described (Kirsch et al., 2010a). Adeno-Cre preferentially infects the lung epithelium and Cre-recombinase acts on the conditional alleles, allowing the mice to develop multiple lung tumors. Tumors become visible on micro-CT, a non-invasive small animal imaging modality, approximately 60 days after Adeno-Cre infection.

### Tissue collection

In order to isolate lung tumors for gene expression and histologic studies, mouse lungs along with other tissues including small intestine, thymus, and spleen were collected at the time of sacrifice. For studies in which bromodeoxyuridine (BrdU) immunohistochemical staining was performed, mice were injected with 5 mg/mL of BrdU 2 h prior to sacrifice to allow for BrdU incorporation to take place. Tissue used for gene expression analysis was stored in RNA*later* (Ambion #AM-7021). Tissue utilized for immunohistochemical analysis was placed in a histology cassette in formalin overnight prior to paraffin embedding. Slides were cut from paraffin embedded tissue to generate unstained slides for immunohistochemical staining.

### RNA isolation and qRT-PCR

RNA was isolated from tissue stored in RNA*later* using a traditional organic solvent precipitation technique followed by RNA cleanup using the RNeasy MinElute Cleanup kit (Qiagen #74204). RNA 260/280 ratios were consistently better than 2.00 and 260/230 ratios were generally better than 1.50. After converting isolated RNA to cDNA using the iScript cDNA Synthesis Kit (Bio-Rad #170-8890), TaqMan probes directed against p53, p21, and PUMA were used to measure gene expression relative to the house-keeping gene GAPDH using a real-time thermal cycler (Bio-RAD, DNA Engine). Student’s *t*-test (two-tailed) was performed to compare relative gene expression among different treatment cohorts.

### Immunohistochemistry

Immunohistochemical staining for BrdU (1:200, Becton Dickinson, Cat #3457580), phospho-histone H3 (1:200, Cell Signaling, Cat#9701), and cleaved-caspase 3 (1:500, R&D Systems, Cat#AF835) was performed on unstained slides cut from formalin-fixed paraffin embedded tissue blocks according to standard immunohistochemistry protocols. Hematoxylin counterstaining was used for all immunohistochemical studies. Analysis of histological specimens was performed by calculating the number of positively staining cells/400× powered field within regions of tumor for each slide. When possible, a two-tailed Student’s *t*-test was performed to compare tumors among different treatment cohorts.

### Radiation treatment

Mice were placed in radiation treatment cradles with lead shielding above and below the thorax to minimize normal tissue injury. Single-dose radiation treatment was delivered using an X-RAD 320 biological irradiator (Precision X-Ray) at a dose output of 320 kV, 10 mA to deliver 11.6 Gy at a dose rate of 69 cGy/min. In the two-fraction arm of the study, radiation was delivered using the same setup and dose rate with 24 h of separation between fractions. To provide an estimate of equivalent dose, we calculated the biologically effective dose (BED) (Fowler, 2010) in two fractions for a single-dose of 11.6 Gy, we estimated that the α/β ratio for lung cancer in mice was 10 and used the following formula for BED or E/α (Fowler, 2006): BED = *E*/α = nd(1 + *d*/α/β), where *E* is the log cell kill from n fractions of *d* grays.

Using an α/β ratio of 10 Gy, the BED for a single 11.6 Gy fraction is 25 Gy_10_. Therefore, the dose required for each of the two fractions to deliver 25 Gy_10_ would be 7.3 Gy. Animals that did not receive any RT still underwent the stress of placement in the radiation treatment cradles for the same length of time as mice that underwent single-dose radiation treatment. Dose rate calibrations were performed by the Duke University Office of Radiation Safety.

### Micro-CT imaging

Computed tomography techniques developed especially for small animal imaging (Micro-CT) allowed us to perform this study. Mice were imaged at 2-week intervals before and after radiation treatment. We used a custom built micro-CT system that has been previously described (Badea et al., 2008). Mice were anesthetized using isoflurane and then were subsequently immobilized in a cradle during CT image acquisition with isoflurane anesthesia via a nose cone delivery system. Projections were acquired using a single tube/detector over a circular orbit of 195° with a step angle of 0.65°. Reconstructions were performed using a commercially available CT reconstruction program (COBRA Exxim, v5.0), with a filtered back projection technique. A resolution of approximately 88 μ/pixel was achieved. The cumulative radiation dose from all three micro-CT scans was 0.3 Gy, which represents about 2% from the 14.6 Gy treatment dose. Using this approach, we have been able to achieve reasonably high-throughput imaging of up to approximately 60 mice in a single day.

### Calculating tumor volumes and generating growth curves

Following CT reconstruction, images were analyzed using an advanced image analysis software suite (Visage Imaging, Amira v5.0). Tumor volumes were calculated by contouring individual tumor areas on each slice of the reconstructed CT scans. In most cases, multiple tumors were contoured for each mouse. The individual assigned to contouring tumor volumes was blinded to the experiment and therefore not aware of mouse genotype or treatment regimen at the time of contouring. Tumors were followed over time before and after radiation treatment and tumor growth rate was determined relative to initial tumor volume calculations. GraphPad Prism software (v5.02) was utilized to perform linear regression and fit growth curves based on tumor volumes from each cohort (*LSL-Kras; p53^FL/FL^* or *LSL-Kras; Ink4a/ARF^FL/FL^*) with 0, 1, and 2 radiation treatments. Student’s *t*-tests were used to analyze differences in relative growth at 14 days after radiation treatment within each mouse genotype.

## Results

### *In vivo* assessment of primary lung cancer growth using micro-CT

**Figure 1:**
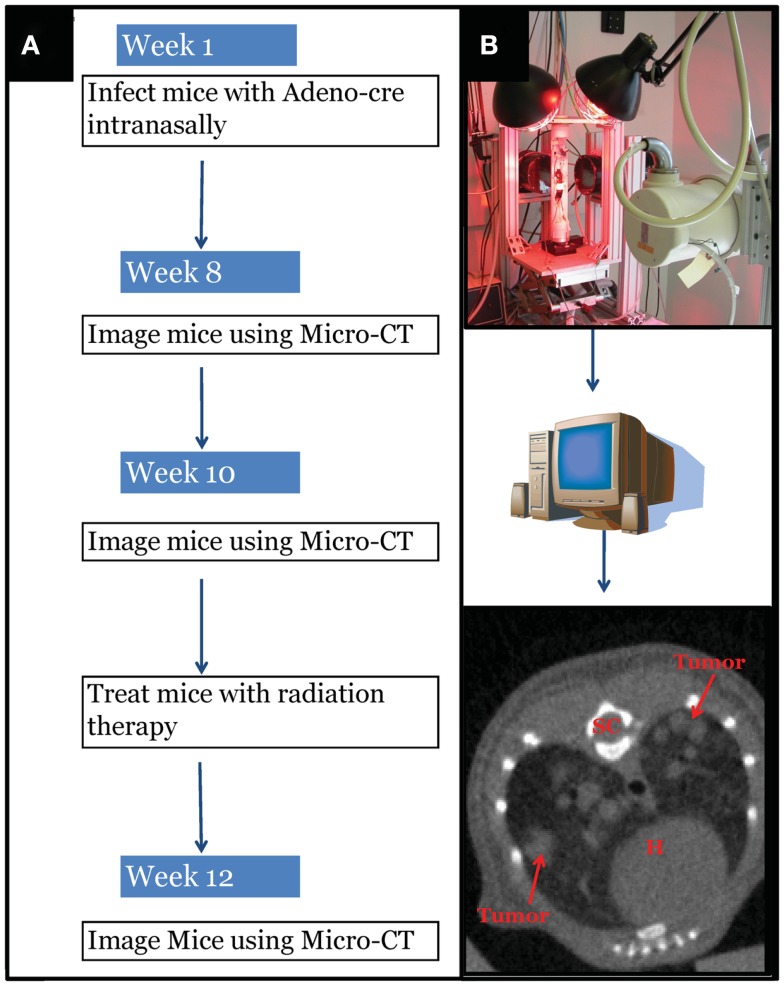
**Experimental design schematic**. **(A)** At around 42 days of age, mice with differing genotypes are infected with an adenovirus expressing Cre-recombinase (Adeno-Cre) via intranasal inhalation. Eight weeks after infection mice develop tumors large enough to be visible by micro-CT imaging. Mice are imaged again at 10 weeks after Adeno-Cre infection to ensure appropriate identification of tumors with growth kinetics suggestive of lung adenocarcinoma. Immediately after a second micro-CT scan, mice undergo whole thorax irradiation with differing treatment regimens. Two weeks after radiation treatment mice are imaged again to evaluate tumor growth delay following radiation treatment. [**(B)** Top Panel] Image of custom built Micro-CT scanner developed at the Duke Center for *In vivo* Microscopy with isotropic resolution of 88 μ. [**(B)** Bottom Panel] Reconstructed image series were utilized to contour tumors to calculate gross tumor volumes and monitor relative growth kinetics. SC, spinal cord; H, heart.

**Figure 2:**
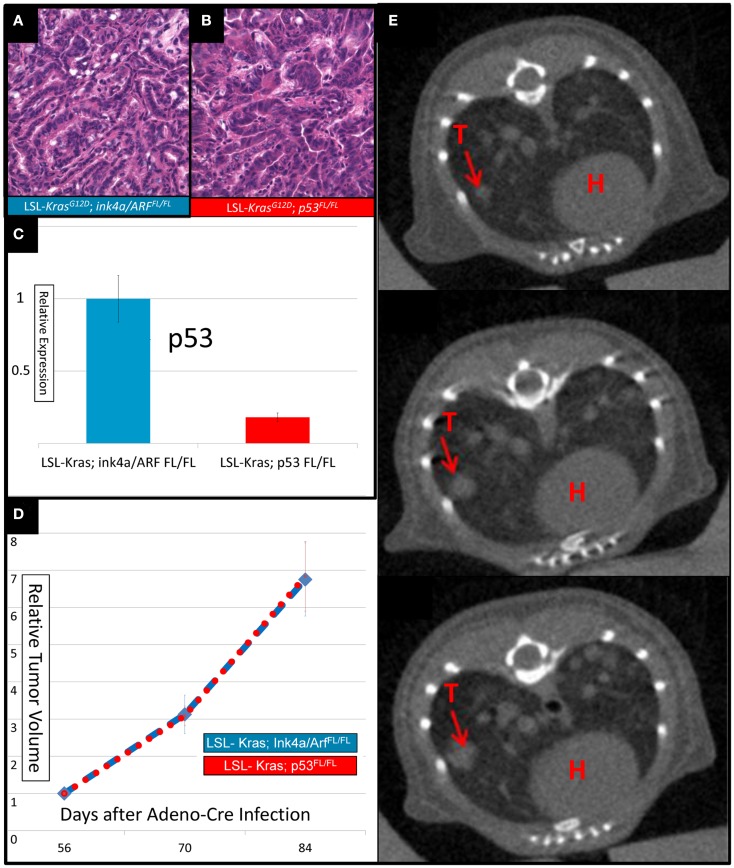
**Lung cancers from mice with conditional mutations in *LSL-Kras; Ink4a/ARF^FL/FL^* and LSL-*Kras*; *p53^FL/FL^* have different levels of p53 expression, but similar growth rates**. Hematoxylin and eosin stained slides of the lung (200×) demonstrate histologically similar adenocarcinomas in **(A)** *LSL-Kras; Ink4a/ARF^FL/FL^* and **(B)** *LSL-Kras; p53^FL/FL^* mice. **(C)** Quantitative RT-PCR shows significantly different levels of p53 expression in lung cancers from *LSL-Kras; Ink4a/ARF^FL/FL^* mice (*n* = 11 tumors) when compared to *LSL-Kras; p53^FL/FL^* mice (*n* = 11 tumors, *P* < 0.0001). **(D)** Relative tumor growth over time with no treatment demonstrates similar growth rates in each tumor cohort at 56, 70, and 84 days following intranasal Adeno-Cre infection. Blue – lung cancers (*n* = 27) from *LSL-Kras; Ink4a/ARF^FL/FL^* mice, Red – lung cancers (*n* = 45) from *LSL-Kras; p53^FL/FL^* mice. **(E)** Serially reconstructed Micro-CT scans at 56, 70, and 84 days after Adeno-Cre infection from a *LSL-Kras; p53^FL/FL^* mouse documents clear and measurable tumor growth over time. T, tumor; H, heart. Error bars – SEM.

Intranasal delivery of Adeno-Cre into *LSL-Kras; p53^FL/FL^* or *LSL-Kras; Ink4a/ARF^FL/FL^* mice led to the development of multiple, aggressive adenocarcinomas in the lungs bilaterally. Primary lung cancers in mice were generated and imaged with micro-CT as previously described (Kirsch et al., 2010a). The mice were imaged with a thoracic micro-CT at 8 weeks after infection (baseline scan), at 10 weeks (1 day before whole lung RT), and at 12 weeks (to assess treatment response) (Figure 1). Lung tumors from both genotypes were histologically similar to previously characterized mouse tumors that are known to resemble human lung adenocarcinomas (Calbo et al., 2005) (Figures 2A,B). We used real-time quantitative RT-PCR to confirm that primary tumors excised from lungs of *LSL-Kras; Ink4a/ARF^FL/FL^* mice (Figure 2C – blue bar) express p53, while tumors from *LSL-Kras; p53^FL/FL^* mice do not (*P* = 0.0001, Figure 2C – red bar). Residual p53 expression in the *LSL-Kras; p53^FL/FL^* tumors is likely from non-epithelial stromal cells or possibly from epithelial cells with an unrecombined p53^FL^ allele (Jackson et al., 2005). Tumor growth rates from both genotypes in the absence of RT (Figure 2D) were similar and the volume doubling time of about 14 days (Figure 2E) was similar to previous reports (Kirsch et al., 2010a; Oliver et al., 2010).

### Tumor response to one or two fractions of RT in lung cancers with deletion of p53 or Ink4A/Arf

**Figure 3:**
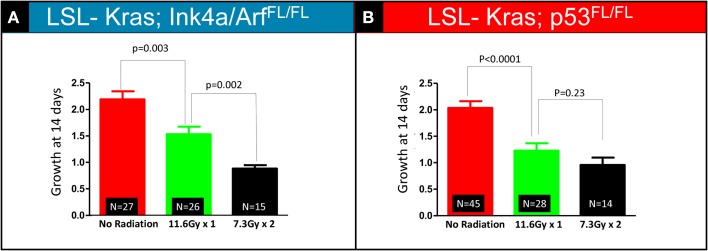
**Quantification of relative tumor volume 14 days after a single 11.6 Gy fraction or two 7.3 Gy fraction radiation treatments**. Lung cancers in **(A)** *LSL-Kras; Ink4a/ARF^FL/FL^* and **(B)** *LSL-Kras; p53^FL/FL^* mice were imaged prior to radiation therapy (Day 0) and 2 weeks after radiation therapy (Day 14). Graphs show the average fold change for the lung tumors at 14 days.**(A)** Lung cancers in *LSL-Kras; Ink4a/ARF^FL/FL^* mice show improved response to two 7.3 Gy fractions separated by 24 h compared to a single 11.6 Gy treatment as measured by micro-CT at 14 days after radiation treatment (*P* = 0.002). **(B)** Lung cancers in *LSL-Kras; p53^FL/FL^* mice respond to a single 11.6 Gy fraction and two 7.3 Gy fractions of radiation therapy with a small difference that is not statistically significant as measured by micro-CT at 14 days after radiation treatment (*P* = 0.23). Error bars – SEM.

The bar graphs depicted in Figures 3A,B show relative volume increase over two time points: time = 0 days (relative tumor volume at the time of RT, 10 weeks after Adeno-Cre infection), and time = 14 days (relative tumor volume 14 days after RT, 12 weeks after Adeno-Cre infection). Relative tumor volume was assessed by normalizing the tumor volume at 14 days after RT to the tumor volume at the time of RT. Lung cancers in *LSL-Kras; Ink4a/ARF^FL/FL^* mice that received a single fraction of 11.6 Gy had decreased growth compared to unirradiated tumors [factor of 1.53 (SEM – 0.03), *n* = 26 tumors vs. factor of 2.19 (SEM – 0.03), *n* = 27 tumors, *P* = 0.003], but two fractions of 7.3 Gy led to an even better response [factor of 0.88 (SEM – 0.01), *n* = 14 tumors, *P* = 0.002, Figure 3A]. Although lung cancers in *LSL-Kras; p53^FL/FL^* mice also responded well to two fractions of 7.3 Gy [factor of 0.96 (SEM – 0.04), *n* = 14 tumors], this was not statistically different than the response to a single fraction of 11.6 Gy (factor of 1.2, *n* = 28 tumors, *P* = 0.23, Figure 3B). Therefore, these results suggest that lung cancers respond differently to one vs. two fractions of RT depending on the genotype of the lung cancer.

### Interrogating the p53 pathway

**Figure 4:**
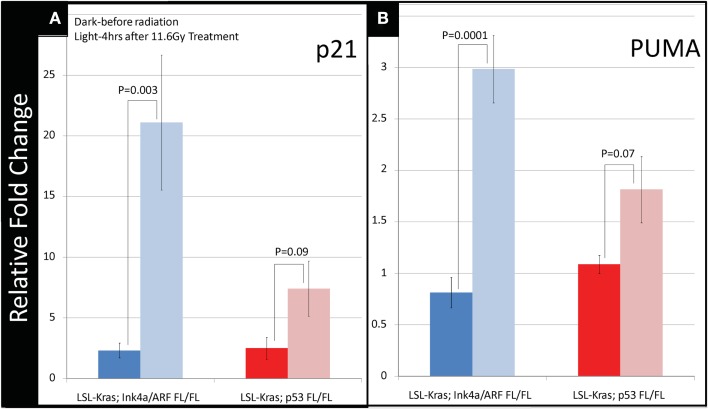
**Downstream p53 effectors, p21 and PUMA, are induced in tumors with wild-type p53 expression following radiation therapy**. **(A)** Lung cancers from *LSL-Kras; Ink4a/ARF^FL/FL^* mice (blue bars, *n* = 6 tumors: no radiation, *n* = 4 tumors: 11.6 Gy × 1) show significantly increased expression of the cyclin-dependent kinase inhibitor, p21, 4 h after radiation treatment, *P* = 0.003. Lung cancers from *LSL-Kras; p53^FL/FL^* tumors (red bars, *n* = 4 tumors: no radiation, *n* = 4 tumors: 11.6 Gy × 1) show non-significant modest increases in p21 expression following radiation treatment, *P* = 0.09. **(B)** Lung cancers from LSL*-Kras; Ink4a/ARF^FL/FL^* mice (blue bars, *n* = 6 tumors: no radiation, *n* = 4 tumors: 11.6 Gy × 1) show significantly increased expression of the p53 upregulated modulator of apoptosis, PUMA, 4 h after radiation treatment, *P* = 0.0001. Lung cancers from *LSL-Kras; p53^FL/FL^* tumors (red bars, *n* = 4 tumors: no radiation, *n* = 4 tumors: 11.6 Gy × 1) show non-significant modest increases in PUMA expression following radiation treatment, *P* = 0.07. Activation of p21 and PUMA within the *LSL-Kras; p53^FL/FL^* tumors may be due to p53 independent activation, non-recombined p53 alleles within the tumor cell population, or p53-dependent activation of stromal cells isolated together with the tumor cells. Error bars – SEM.

To investigate how p53-mediated signaling influences the radiation response of lung tumors, we examined the mRNA expression of p53 targets, which regulate p53-mediated cell-cycle arrest and apoptosis, in lung tumors after a single fraction of 11.6 Gy whole lung RT. Primary tumors of each genotype were excised from the lungs 4 h after RT, and the mRNA levels of p53 transcriptional targets p21 and PUMA were measured by quantitative reverse transcription-polymerase chain reaction (qRT-PCR). In tumors with WT p53 (*LSL-Kras; Ink4a/ARF^FL/FL^*) a nearly 20-fold increase in p21 mRNA levels was noted after RT (*P* = 0.003, Figure 4A – blue bars). Tumors from *LSL-Kras; p53^FL/FL^* mice showed significantly lower levels of p21 activation after RT (*P* = 0.09, Figure 4A – red bars). Similarly, RT-induced PUMA mRNA expression was robust in tumors from *LSL-Kras; Ink4a/ARF^FL/FL^* mice, but not in tumors from *LSL-Kras; p53^FL/FL^* mice (*P* = 0.0001, Figure 4B – blue bars). Thus, these data indicate that p53-mediated DNA damage response is significantly more active in *LSL-Kras; Ink4a/ARF^FL/FL^* tumors as compared with *LSL-Kras; p53^FL/FL^* tumors.

**Figure 5:**
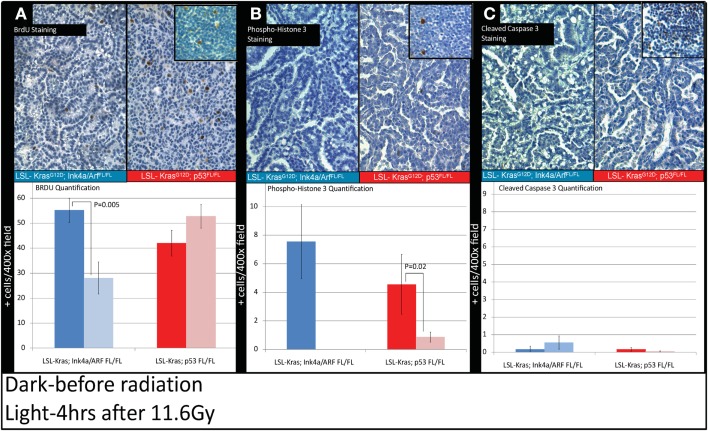
**Immunohistochemistry of lung cancers shows an intact radiation-induced G1 cell-cycle arrest in lung cancers from *LSL-Kras; Ink4a/ARF^FL/FL^* mice, but not from *LSL-Kras; p53^FL/FL^* mice**. Immunohistochemistry of sections of lung from mice with lung cancer with the indicated genotype was performed for: **(A)** Bromodeoxyuridine (BrdU). **(B)** Phospho-Histone H3. **(C)** Cleaved-Caspase 3. Top Panels show representative immunohistochemistry for lung tumors in *LSL-Kras; Ink4a/ARF^FL/FL^* and *LSL-Kras; p53^FL/FL^* mice 4 h after radiation treatment with 11.6 Gy (200×, brown-antibody immunostaining, blue – hematoxylin). Inserts show positive control staining for each antibody within unirradiated (BrdU or phospho-histone H3) and irradiated (cleaved-caspase 3) thymus. Bottom panels show quantification of immunohistochemical staining which was performed by counting the number of positively stained cells/random 400× powered field within the lung tumor parenchyma. All analysis was done in a blinded fashion to treatment group and genotype. Error bars – SEM.

Because the induction of cyclin-dependent kinase inhibitor p21 contributes to the G1 cell-cycle arrest after irradiation (Kastan and Bartek, 2004), we evaluated the progression of cell-cycle after RT in the tumors from *LSL-Kras; Ink4a/ARF^FL/FL^* and *LSL-Kras; p53^FL/FL^* mice by the labeling index of BrdU, which is a thymidine analog that is incorporated into replicating DNA. Immunostaining of the lungs with an antibody against BrdU (Figure 5) revealed that tumors from *LSL-Kras; Ink4a/ARF^FL/FL^* mice had significantly decreased BrdU uptake 4 h after RT (*P* = 0.005, Figure 5A), as compared with unirradiated tumors, indicating the presence of an intact G1 cell-cycle checkpoint. Thus, lung cancers with an intact p53-dependent induction of p21 appear to undergo a radiation-induced G1 cell-cycle arrest*in vivo*. In contrast, lung tumors from *LSL-Kras; p53^FL/FL^* mice incorporated high levels of BrdU after irradiation, indicating that these tumors lacked an intact G1 cell-cycle checkpoint (Figure 5A). Interestingly, immunohistochemical staining for phospho-histone H3, which is present in cells during mitosis, revealed a significant reduction in tumors from both genotypes after RT (Figure 5B). Finally, cleaved-caspase 3 staining was performed to evaluate the extent of radiation-induced apoptosis. At 4 h after RT, apoptosis was an uncommon event in both tumor types (Figure 5C).Taken together, these assays provide functional data to demonstrate that only in primary lung tumors from *LSL-Kras; Ink4a/ARF^FL/FL^* mice, which retain p53, is the radiation-induced p53 response functionally intact.

## Discussion

In this study, we utilized genetically engineered mouse models of lung cancer to study the treatment response of one vs. two fractions of radiation in lung adenocarcinomas harboring different mutations in tumor suppressor genes. We have shown that lung cancers in *LSL-Kras; Ink4a/ARF^FL/FL^* mice maintain the ability to induce the p53 transcriptional target p21 and to activate the G1 cell-cycle arrest. In contrast, we find that lung cancers in *LSL-Kras; p53^FL/FL^* mice, which lack WT p53, have an impaired induction of p21 after irradiation and fail to induce a G1 cell-cycle arrest. Our inability to appreciate radiation-induced pre-mitotic apoptosis in the tumor cells 4 h after RT in either of the two mouse models of lung cancer may be because NSCLC tumors, like many other carcinomas, do not respond to radiation damage with a robust apoptotic response (Gudkov and Komarova, 2003). This factor is likely the reason for a lack of p53 mediated radiation-induced apoptosis in either of the two tumor types. Alternatively, it is also possible that the time point (4 h after RT) we chose in this study is not appropriate to detect cell death in these tumors after radiation. Lung cancers with intact p53 in *LSL-Kras; Ink4a/ARF^FL/FL^* mice had an improved response to two fractions of 7.3 Gy vs. a single fraction of 11.6 Gy. In contrast, lung cancers lacking p53 in *LSL-Kras; p53^FL/FL^* mice did not have a statistically significant difference in the response to these two treatment regimens. These results suggest that tumor genotype can affect the radiation response of primary cancers to one vs. two fractions of RT.

This study has some limitations in recapitulating the treatment of human lung cancer with RT. With inhalation of Adeno-Cre, multiple tumors develop in a single mouse, which is not common in patients diagnosed with NSCLC. We also utilized whole lung RT, which may impact tumor growth differently than the relatively small volume RT fields used in the clinic to treat lung cancer. Additionally, fraction sizes of 7.3 and 11.6 Gy are not standard doses used to treat human lung cancer. Finally, while tumorigenesis was initiated by recombination to activate LSL-Kras and delete both alleles of either p53 of Ink4a/ARF, it is possible that some tumors did not undergo all recombination events. In addition, it is likely that the tumors developed additional random oncogenic mutations as they grow means heterogeneity exists in each of the cohorts.

Our experimental approach cannot differentiate whether differences in radiation response between the two cohorts is due to loss of p53 function in the *LSL-Kras; p53^FL/FL^* tumors, loss of Ink4A/Arf in the *LSL-Kras; Ink4a/ARF^FL/FL^* tumors, or a contribution of the presence or absence of both gene mutations. Because the genetic backgrounds of the *LSL-Kras; p53^FL/FL^* and *LSL-Kras; Ink4a/ARF^FL/FL^* mice are not the same, we have not made a direct comparison of tumor response for each treatment across the two genotypes. However, the data from Figure 3 suggests that lung cancers from both genotypes appear to have a similar response to 7.3 Gy × 2, but there may be an increased response of lung cancers in *LSL-Kras; p53^FL/FL^* mice to a single-dose of 11.6 Gy. This could be one reason why lung cancers in this genotype did not demonstrate a statistically significant different response to 11.6 Gy × 1 vs. 7.3 Gy × 2 treatments.

Recently, we have demonstrated that certain normal tissues lacking p53 have an increased sensitivity to radiation exposure. For example, mice in which p53 is specifically deleted in gastrointestinal (GI) epithelial cells are sensitized to the radiation-induced GI syndrome (Kirsch et al., 2010b). Others have also reported that mice lacking p53 are sensitized to the radiation-induced GI syndrome (Komarova et al., 2004; Leibowitz et al., 2011). In addition, we have shown that mice lacking p53 specifically in endothelial cells are sensitized to radiation-induced cardiac injury (Lee et al., 2012). Because mice lacking p21 are also sensitized to the radiation-induced GI syndrome and to radiation-induced cardiac injury (Komarova et al., 2004; Kirsch et al., 2010b; Leibowitz et al., 2011; Lee et al., 2012), it is conceivable that lung cancers lacking p53 are less likely to respond to fractionated RT as compared to a large single-dose of RT. This difference in response may be due in part to a deficiency in the induction of p21 expression and activation of the G1 cell-cycle checkpoint. Therefore, based on these results we hypothesize that the p53 pathway and its ability to induce a G1 cell-cycle arrest in tumor cells (Figure 5A) may play a role in the differential response of lung cancers to one or two fractions of RT.

We selected the two radiation regimens (11.6 Gy × 1 vs. 7.3 Gy × 2) based on a calculation for the BED and used a commonly acceptedα/β ratio of 10 Gy for lung cancer. The difference in growth delay that we observed in lung cancers at 14 days between the two regimens in the two genetic variants of the NSCLC raises the intriguing possibility that the α/β ratio may not be equivalent in the two mouse models of NSCLC. While more data points will be required to formally define the α/β ratio in both mouse models of lung cancer, we believe that such experiments have the potential to provide a mechanistic basis at the genetic level for differences in the α/β ratio.

In summary, we have investigated the impact of different gene mutations in lung cancer on the response to one or two fractions of radiation therapy using advanced mouse models of primary lung cancer. This system has advantages, albeit at a cost, to traditional xenograft or even knockout mouse models. Tumors form within the native tumor environment (lung parenchyma) and tumor formation is temporally controlled by the initiation of tumorigenesis with Adeno-Cre infection during adulthood. Furthermore, expression of mutant alleles driving tumorigenesis occurs from the endogenous gene promoters at physiologic levels. Others have shown that such GEMMs of lung cancer may more faithfully predict the response of human lung cancers to systemic therapy (Singh et al., 2010; Chen et al., 2012). Therefore, we anticipate that these mouse models will similarly be useful for investigating radiation biology (Kirsch, 2011).

## Conflict of Interest Statement

The authors declare that the research was conducted in the absence of any commercial or financial relationships that could be construed as a potential conflict of interest.
